# Protein Fibrillation under Crowded Conditions

**DOI:** 10.3390/biom12070950

**Published:** 2022-07-06

**Authors:** Annelise H. Gorensek-Benitez, Bryan Kirk, Jeffrey K. Myers

**Affiliations:** 1 Department of Chemistry and Biochemistry, Colorado College, Colorado Springs, CO 80903, USA; 2 Department of Biology, Davidson College, Davidson, NC 28035, USA; brkirk@davidson.edu; 3 Department of Chemistry, Davidson College, Davidson, NC 28035, USA

**Keywords:** aggregation, amyloid fibril, excluded volume, molecular crowding, neurodegenerative disease, molecular crowding, osmolyte, polyol, protein fibrillation, proteopathy, synthetic polymer, viscosity

## Abstract

Protein amyloid fibrils have widespread implications for human health. Over the last twenty years, fibrillation has been studied using a variety of crowding agents to mimic the packed interior of cells or to probe the mechanisms and pathways of the process. We tabulate and review these results by considering three classes of crowding agent: synthetic polymers, osmolytes and other small molecules, and globular proteins. While some patterns are observable for certain crowding agents, the results are highly variable and often depend on the specific pairing of crowder and fibrillating protein.

## 1. Introduction

The processes that lead to protein aggregates are under intense scrutiny, particularly those which result in the formation of amyloid fibrils (long, insoluble inclusions that are rich in β-strand structures). Amyloid fibrils have a functional role in many organisms [1], and have been implicated in the pathology of many human diseases, including increasingly widespread neurodegenerative diseases, such as Alzheimer’s and Parkinson’s [2]. Additionally, many of the proteins and peptides unrelated to human disease fibrillate under laboratory conditions; controlled fibrillation may have important bioengineering applications. Thus, fibrillation is an inherent property of polypeptides and is worthy of study, even in the absence of a role in disease or biological function.

Understanding diseases where fibrillation is prominent requires an appreciation of the aggregation of proteins under physiological conditions, conditions that are poorly represented by dilute aqueous solutions. In the cells and extracellular matrices, the proteins fold and misfold in a crowded environment, surrounded by a complex (and nonrandom) mixture of other solutes [3,4]. Ideally, fibrillation would primarily be studied in living organisms [5,6,7,8,9,10], however, measuring the kinetics of fibrillation in the cells poses obvious technical challenges. Instead, researchers have attempted to mimic the crowded environment of cells in vitro, via crowding agents [11]. The crowded solutions may also be used to tease out the mechanistic details of the fibrillation process, since the critical steps along the pathway toward the fibril involve an association of protein chains, and molecular crowding generally favors such an association.

The synthetic polymers, including the carbohydrate polymers, Ficoll and dextran, polyethylene glycol, and polyvinylpyrrolidone, among others, have been used. These crowders have multiple effects on the stability, folding, structure and misfolding of proteins, arising from excluded volume, viscosity, weak interactions between the protein of interest and the crowding agent, and changes in solvation [12]. The excluded volume alone cannot explain all of the observed effects [13], and these varied influences complicate the interpretation of the data. Generally, crowding favors the fibrillation of disordered proteins, such as α-synuclein, while disfavoring the fibrillation of oligomeric proteins, such as insulin. However, the results vary depending on the protein and type of crowder.

We summarize the results of the fibrillation of proteins under crowded conditions, and attempt to make sense of those data from two perspectives. First, are there common mechanisms of fibrillation? Second, are certain crowding agents preferable for mimicking intracellular conditions?

We dedicate this review to the memory of Christopher M. Dobson, who made seminal contributions to the field of protein science and whose work continues to influence many. Dobson’s exploration of the effects of crowding on aggregation began in the late 1990s and continued through the 2010s [14]. Several of his works will be discussed in the text, and of particular interest is one study, including a novel method to measure the elongation rate of fibrillation using quartz crystals [15]. Dobson’s 2003 review remains an excellent introduction to protein misfolding [16], and did much to bring wider attention to what was then an underappreciated, emerging field of great practical importance.

## 2. An Overview of Macromolecular Crowding

Many studies of protein are carried out in dilute buffered solutions. However, the biological milieu can be very crowded. For example, the cytoplasm of a typical cell can contain upwards of 300 g/L proteins alone [17]. The macromolecular crowding effect exerted by the cellular interior has the potential to alter not only the individual protein properties [18], but the interrelationship between the proteins (for example, the liquid–liquid phase separation of the proteins in cells [19]).

Historically, theories of macromolecular crowding treated proteins as hard spheres that did not interact, except through steric repulsions. The proteins take up volume that is then excluded from the neighboring macromolecules, resulting in an entropic compaction of proteins and the adoption of the most compact (usually the native) state [18,20,21,22,23]. Volume exclusion was shown to affect protein stability, folding kinetics [24], enzyme activity [25,26], and aggregation [27,28]. Macromolecular crowding is of utmost importance for understanding the proteopathies and protein aggregation. Many of the protein misfolding disorders, such as Alzheimer’s and Parkinson’s Disease, occur primarily with age. One idea is that the cells become dehydrated, and the effective concentration of proteins in the cell increases, leading to increased protein fibrillation [29]. Understanding how the fibrillation is affected by macromolecular crowding can help us understand the disease and ultimately design better therapeutics.

Since the nascence of the macromolecular crowding field over forty years ago [18,30,31,32,33], an additional layer has been added to our understanding: enthalpically-driven chemical interactions between the proteins and crowders, including electrostatic interactions, hydrogen bonding, and hydrophobic interactions [34]. If the chemical interactions are repulsive, they are additive to excluded volume effects, but if they are attractive, they counteract the volume exclusion. The weak chemical interactions have been demonstrated as modulating protein stability [35,36,37,38], folding kinetics [39], and activity [40,41].

## 3. Synthetic Polymers

**Figure 1:**
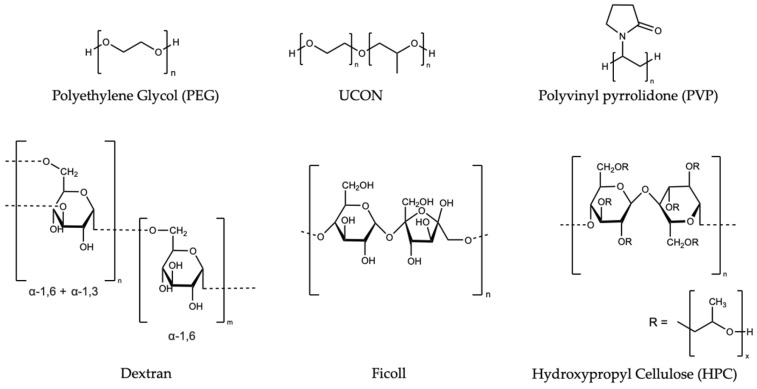
Structures of synthetic polymers referenced in this Review.

**Figure 2:**
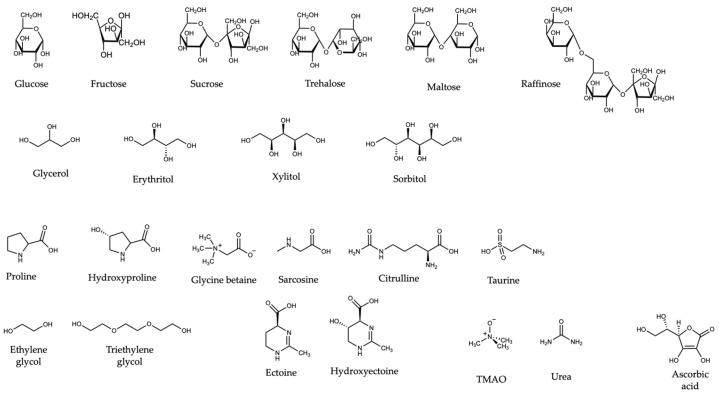
Structures of osmolytes and small molecules referenced in this Review.

Historically, synthetic polymers, including the sugar-based polymers Ficoll and dextran [24,38,39,41], polyethylene glycol (PEG) [42], and polyvinylpyrrolidone (PVP) [43,44], were used to represent the cellular environment. A summary of the commonly used polymers, their abbreviations, and average molecular weights are included in Table S1, Supplementary Materials. These polymers affect the proteins via both steric repulsion and weak chemical interactions. The effects of their monomers, some of which are osmolytes [45], can be used to contextualize and decode the effects of the polymers [37,38,39]. The bond-line structures of commonly used synthetic polymers and osmolytes are presented in Figure 1 and Figure 2, respectively.

Ultimately, the synthetic polymers are not the best representation of cells [36,37]. Another option is to use reconstituted cytosol, lysates [36,46,47] or model proteins, such as hen egg white lysozyme (HEWL or lysozyme), and bovine serum albumin (BSA) [38,48] as the crowders. However, these biopolymers still fall short of accurately replicating the cellular interior [38]. Both synthetic and physiologically relevant crowders pose challenges not seen in dilute solution experiments, including increased solution viscosity, high background, and decreased signal quality due to interactions between crowders and test proteins [39,41]. The effects of crowding on protein structure and function have been probed in living cells, but in-cell experiments pose many of the same challenges, with the additional concern of cell leakage [49,50,51,52,53,54]. References and results from fibrillation experiments under crowded solutions are listed in Table 1, Table 2 and Table 3. Table 1 details the effects of synthetic polymers, Table 2 of small molecule osmolytes, and Table 3 of protein crowders. We include a version of Table 1 and Table 2 organized by protein in the Supplementary Materials (Tables S2–S5 Supplementary Materials).

## 4. Synthetic Polymers and Protein Fibrillation

In their 2010 *Journal of the American Chemical Society* publication [15], Dobson and coworkers studied the effects of the synthetic PEG 200,000, dextran 200, and the dextran monomer and osmolyte, glucose (Refer to Table S1, Supplementary Materials, for the average molecular weight of these polymers). This study used pre-nucleated fibrils, enabling measurements that exclusively probe the elongation rates. They interpreted their results using the framework of scaled-particle theory [55,56,57], which posits that the excluded volume effects decrease with the increasing particle size. The limits of scaled particle theory to analyze the crowding effects on fibrillation were acknowledged, as the parameters of the study can only account for the fibril elongation. As expected, the analysis of fibrillation in the complex cellular matrix has seen limited success [58]. The effects of PEG 200,000 and dextran 200 are considered here.

A variety of amyloid-prone proteins of different sizes were used, because scaled particle theory predicts that the fibrillation rates increase with the increasing hydrodynamic radius of the precursor protein. The proteins include the globular proteins, lysozyme and insulin, and proteins lacking a well-defined tertiary structure, including the SH3 domain of the phosphatidyl-inositase-3-kinase (SH3), α-synuclein, and the β-domain of insulin at pH 2. The amyloid elongation was measured as a function of the increasing dextran 200 ranging from 0–60 g/L. Dextran 200 accelerates the relative fibrillation elongation rates of all of the proteins, and this enhancement increases with the increasing hydrodynamic radius of the test protein. As the trends with an increasing extent of acceleration as a function of the protein hydrodynamic radius is consistent with scaled particle theory, these affects are attributed to the volume exclusion by dextran 200. PEG 200,000 was also found to accelerate the relative rate of elongation of insulin, and to a greater extent than dextran 200. The promotion of fibrillation in the synthetic polymers is also observed in several other studies [42,48,59,60].

These findings agree with the pioneering work of Uversky and coworkers, which began with synthetic polymers and α-synuclein, the protein implicated in Parkinson’s Disease [48]. The crowders’ identity, size, and concentration were considered. PEG, Ficoll, and dextran promote fibrillation by increasing the rate and decreasing the lag time. Of the three types of polymers, PEG is the most drastic accelerant. The PEGs with the largest molecular weight (3350 Da) exert stronger effects than the smaller PEGs (200 Da, 400 Da, 600 Da). The fibrillation is increasingly accelerated as the PEG 3350 concentration increases from 25 to 150 mg/mL.

While PEG most effectively promotes α-synuclein fibrillation, the effects of dextran 138, Ficoll 70, and Ficoll 400 were also considered (Refer to Table S1, Supplementary Materials, for average molecular weight). Ficoll 400 is slightly more effective than Ficoll 70 at increasing the fibrillation rate and decreasing the lag time, but both are more effective than dextran 138. Ultimately, the authors observe the modulation of the fibrillation depends on the identity of the polymer, and within a single type of polymer, the fibrillation increases with increasing size and concentration. The authors attribute these effects to excluded volume, and eliminated increased solution viscosity as an explanation, as polymers decreased the lag time of the reaction.

A subsequent publication expanded the exploration to a variety of proteins, including *S*-carboxymethyl lactalbumin, human insulin, bovine core histones, and human α-synuclein [59]. Whereas the proteins selected by Dobson and coworkers vary in degrees of disorder, these proteins additionally vary in oligomeric state. Consistent with other studies of α-synuclein [15,48], the polymers such as Ficoll 70 and PEG 3500 accelerate the fibrillation of disordered proteins, namely α-synuclein and *S*-carboxymethyl lactalbumin, by increasing the fibrillation rate and decreasing the lag time. The proteins that occupy an oligomeric state before fibrillation, such as bovine core histones, see hindered fibrillation in the presence of PEG 3500. Another example, human insulin, illustrates the complexity of crowding effects, as it can adopt both a monomeric and hexameric state under experimental conditions. The observations for monomeric insulin in the presence of PEG 3500 and Ficoll 70 are consistent with observations for α-synuclein and *S*-carboxymethyl lactalbumin at a neutral pH; where insulin is a hexamer the polymers slow fibrillation, increasing the lag time, because the oligomer must first dissociate and undergo a structural change. For the oligomeric proteins, therefore, the polymer crowders hinder fibrillation, probably because the crowded conditions favor the formation of the native oligomer.

In subsequent publications, the Uversky group probed the role of polymer morphology and flexibility [12]. The authors asserted that the effect of crowding depends on a test protein’s shape, size, and degree of order. The commonly-used synthetic polymers, dextran and Ficoll, are compact and flexible polysaccharides. However, most biopolymers in the cell—nucleic acids, proteins, etc.—are more rigid. The cellulose-derived polymers, hydroxypropyl cellulose (HPC) 100, 370, and 1000 were chosen to represent the effects of the more rigid polymers, while the dextrans 100, 250, and 500 were used to represent the more commonly used flexible polymers (See Figure 1 for a structural comparison; Table S1, Supplementary Materials, for average molecular weight). Unsurprisingly, the two types of polymers exhibit opposite effects on the proteins with different characteristics. The dextrans inhibit the proteins that form stable oligomers before or during fibrillation, including insulin at pH 7.5 and α-lactalbumin. By contrast, the dextrans accelerate the fibrillation of the disordered proteins, α-synuclein and histones. Modest effects in either direction are seen with the monomeric globular proteins, lysozyme and insulin, at pH 2.5. This trend indicates that the dextrans operate by excluded volume, favoring the most compact form of the test protein. The HPCs of all sizes, on the other hand, hindered fibrillation for all of the proteins—with the exception of histones, which may be due to the inability of histones to fold under the assay conditions.

Of particular interest were the contributions from excluded volume, viscosity, and weak interactions (such as electrostatic interactions, dipole–dipole interactions, and hydrogen bonds). To parse the effects of the excluded volume and viscosity, dextran 500 and Ficoll 400 (which has a similar size but a higher density and lower viscosity) were used as the crowders. These two polymers should exert roughly the same excluded volume, based on their close average molecular weight. Both dextran 500 and Ficoll 400 also hindered α synuclein and monomeric insulin fibrillation. However, Ficoll 400 did so more effectively, indicating that the excluded volume effects of dextran are likely counteracted by viscosity. However, an inhibition of fibrillation was still seen in the solutions of relatively low viscosity, suggesting the contribution of weak chemical interactions between the proteins and polymers [12].

Next, the role of polymer hydrophobicity was probed [61]. Most of the commonly-used synthetic polymers are hydrophilic. UCON 5400 (1:1 copolymer of ethylene- and propylene- glycol, Figure 1) is structurally similar to PEG 4400 but has an extra methyl group on every other unit. The additional methyl group on this polymer, in contrast to PEG, provides an excellent comparison of hydrophobicity. The effects on the secondary structure and intrinsic fluorescence quenching for 10 proteins of varying size, degree of structure, and oligomeric state were probed, while the fibrillation kinetics and morphology were explored. Circular Dichroism (CD), 8-anilonapthalene-1-sulfonate (ANS) fluorescence, and acrylamide quenching demonstrate that, while PEG and UCON do not affect the protein secondary structure, they change the solvent accessibility. Ultimately, UCON is more effective at unfolding the test protein than PEG. As with previous studies, PEG enhances the fibrillation of α-synuclein and monomeric insulin, decreasing the lag time and increasing the elongation rate. UCON, however, inhibits the fibrillation of insulin, and further analysis of the samples with a scanning electron microscopy (SEM) revealed UCON instead promotes the oligomerization of α-synuclein. Uversky and colleagues attributed the PEG effects to excluded volume, while the UCON effects were suggested to arise from changes in the solvent properties.

The enhanced fibrillation of α-synuclein and other disordered proteins in synthetic polymers, as seen in Uversky’s early studies [42,48,62] and Dobson’s publication [15], is observed in other instances. Shtilerman and colleagues observed size-dependent reduction of lag times; PEG 3350 exerts the most dramatic effect, followed by dextran 70 and Ficoll 70, while a similar trend was observed with PEGs of varying sizes. [60]. In another study, β-lactoglobulin fibrillation is accelerated—specifically the lag time decreases and the fibrillation rate increases—in Ficoll 70 and PEG 400 (400 Da), 8000 (8000 Da), and 20,000 (2000 Da). The effect is more pronounced with an increasing size and concentration, and therefore is attributed to excluded volume [63]. Wu and colleagues observed that Ficoll 70 and dextran 70 enhanced the fibrillation of human Tau protein, with dextran 70 exerting stronger effects [64].

A fibrillation-prone fragment of Tau protein was the subject of a study by Ma et al. [62], who also examined a cohort of other pathogenic, fibrillation-prone proteins, including human prion protein (PrP), and its variants E196K and D178N, the A4V SOD1 which is implicated in ALS [65]. In addition, rabbit PrP and hen egg white lysozyme were considered, both of which are not pathogenic. Ficoll 70, dextran 70, and PEG 2000 promote the fibrillation of both the unphosphorylated versions of the Tau fragment, from 50 g/L to 200 g/L. Similarly, crowding with Ficoll 70 and Ficoll 400 accelerates the fibrillation of the human prion protein, and variant, while dextran 70 and PEG 2000 enhance the SOD1 fibrillation.

The authors observed that the phosphorylated Tau protein fragment, which is associated with the onset of Alzheimer’s Disease, does not fibrillate in dilute solution. However, it fibrillates in the presence of Ficoll 70 and dextran 70, which the authors attribute to one of two explanations. The first possibility is that the phosphorylated Tau is more likely to fibrillate in a crowded environment, while the second is that crowding works to counteract the retardation initiated by phosphorylation.

Conversely, the authors found that the macromolecular crowding promote the fibrillation of the non-fibrillation-prone proteins, rabbit PrP and hen egg white lysozyme, at 100 g/L but hindered the fibrillation at 200 and 300 g/L. Whereas Dobson and coworkers saw an acceleration in the presence of crowders, regardless of the protein’s structure; thus, these authors concluded that the macromolecular crowding effects vary depending on the protein and crowder selected. Proteins that are prone to fibrillate will do so under crowded, cell-like conditions, as crowding stabilizes the aggregates or multimers along the path to aggregation. For proteins that are not aggregation-prone, such as lysozyme and rabbit Prp, the authors hypothesized that competition between the stabilization of aggregates and of the folded, native state, come into play, which led to the disparate results at 100, 200, and 300/gL crowder.

A recent study by Biswas and coworkers [66] demonstrated that the polymers of differing sizes can have opposing effects on the α-synuclein fibrillation. Specifically, with in vitro experiments, the lowest molecular weight PEG, PEG 600, hindered fibrillation by increasing the lag time. PEG 1000 increased the lag time, and the fibrillation rate, but the increase in lag time was more dramatic, hindering fibrillation. The higher-mass PEGs, PEG 4000 and PEG 12,000, both led to decreases in the lag time and in the fibrillation rate, with a more drastic reduction seen with PEG 20000. For the larger PEGs, the decrease in lag time was more dramatic, therefore promoting fibrillation. Overall, the promotion of fibrillation by the higher molecular weight PEGS was consistent with the findings of Dobson and coworkers [15], as well as other earlier studies [42,48]. Next, the authors explored how the presence of PEG 6400 and 8000 affected the fibrillation of the α-synuclein A53T in yeast cells; they found that the effect was opposite of that found in vitro. In living cells, the addition of these two PEGs hindered fibrillation. However, the authors found that the concentration of soluble α-synuclein in the cells was higher with the PEGs added than without, suggesting that in the cells, PEG may be acting to solubilize the α-synuclein monomers, and therefore working to counter aggregation.

The globular protein hemoglobin was the subject of a thorough study by Siddiqui and Naeem [67]. Specifically, the authors investigated the effects of PEG 4000, PEG 6000, and dextran 70 on hemoglobin fibrillation, and then used isothermal titration microcalorimetry (ITC) to quantify any weak interactions between the protein and the polymers. Hemoglobin, on its own under the conditions selected in the paper, did not undergo fibrillation. PEG 4000, 6000, and dextran 70, at a concentration of 200 g/L, were all found to promote hemoglobin fibrillation, with dextran 70 exhibiting the strongest effects. From the ITC experiments, it was determined that the specific binding of hemoglobin by polymers was not responsible for this change. The characteristics of the hemoglobin fibrils, however, were shown to be different, depending on the crowder. The fibrillation experiments with Congo Red demonstrated that PEG 4000 promotes the formation of protofibirils, while the fibrils were formed in the presence of PEG 6000 and dextran 70, the results of which were confirmed using morphological studies of hemoglobin aggregates using SEM. The authors then expanded upon the study by determining the effects of such aggregation on living cells, specifically, of human peripheral blood cells (PBMCs). The formation of hemoglobin aggregates in the presence of crowders reduced cell viability, manifested by an increase in lipid peroxidation, a decrease in mitochondrial membrane potential, and an increase in the number of necrotic cells relative to apoptotic cells. Additionally, the cells with aggregating hemoglobin in the presence of crowders showed increased DNA damage. All of these effects were attributed to an increase in the radical oxygen species from oxidative stress. Ultimately, the increase in the protein fibrillation due to the presence of the macromolecular crowding was demonstrated to have potentially devastating physiological impacts. This is of particular concern to the elderly, where proteopathies occur more frequently, as the cells shrink and dehydrate with age.

Dobson’s work, and many efforts, show that crowding by synthetic polymers accelerates fibrillation. However, several studies report mixed results: polymers either hinder the fibrillation, or have no effect. For example, Ficoll 70 and dextran 70 stabilize the native state of the β-sheet rich protein, bovine carbonic anhydrase, leading to decreased fibrillation rates and fewer aggregates [28]. Kong and Zeng found that the effects on lysozyme fibrillation depend on whether the fibrils are pre-seeded [68]. When the lysozyme fibrils are not pre-seeded, adding PEG slows the fibrillation. This trend is reversed when the lysozyme is pre-seeded, with PEG accelerating the fibrillation, suggesting that crowding stabilizes the intermediate oligomers instead of the fibrils, while the pre-formation of anchors for fibrils encourages fibrillation. The authors saw the effects on lysozyme fibrillation, even at low PEG concentrations of 10–20 g/L. The presence of the effects at such small concentrations of polymer crowders led the authors to conclude that the chemical interactions are a key factor.

The Winter group assessed the effects of a variety of crowding agents on human islet amyloid polypeptide (hIAPP), which is implicated in Type 2 Diabetes. Contrary to the other crowding agents in the study, Ficoll 70 does not affect the fibrillation of hIAPP. Low concentrations of dextran 70 (10–20%) also do not affect the fibrillation, while high concentrations of dextran (30–40%) caused a loss in the sigmoidal shape of the data and a lengthened elongation time, which the authors suggest is the result of a more complex fibrillation mechanism. The slight differences in these effects were attributed to viscosity, as dextran exhibits a higher viscosity than Ficoll [69].

**Table 1:** Effects of synthetic polymers on protein fibrillation.

Polymer	Test Protein	Effect
Ficoll 70	α-synuclein	Promotes fibrillation [42,48,59,60]
	Tau Protein	Promotes fibrillation [64]
	Insulin, pH = 2	Promotes fibrillation [59]
	Insulin, pH = 7.5	Hinders fibrillation [59]
	Bovine Carbonic Anhydrase	Hinders fibrillation [28]
	hIAPP	No effect [69]
	Unphosphorylated Tau 244–372	Promotes Fibrillation [65]
	Phosphorylated Tau 244–441	Promotes Fibrillation [65]
	Human PrP	Promotes Fibrillation [65]
	Human PrP E196K	Promotes Fibrillation [65]
	Human PrP D178N	Promotes Fibrillation [65]
	Rabbit PrP	Hinders Fibrillation [65]
	HEWL	Hinders Fibrillation [65]
	α-lactalbumin	Promotes Fibrillation [59]
Ficoll 400	α-synuclein	Promotes fibrillation [42,48,59]
	Human PrP	Promotes Fibrillation [65]
	Human PrP E196K	Promotes Fibrillation [65]
	Human PrP D178N	Promotes Fibrillation [65]
Dextran 70	Human Tau protein, 50–100 g/L	Promotes fibrillation [64]
	Human Tau protein, 150 g/L	Hinders fibrillation [64]
	α-synuclein	Promotes fibrillation [60]
	Bovine Carbonic Anhydrase	Hinders fibrillation [28]
	hIAPP, 10–20%	No effect [69]
	hIAPP, 30–40%	Hinders fibrillation [69]
	Hemoglobin	Promotes fibrillation [67]
	Unphosphorylated Tau 244–372	Promotes fibrillation [65]
	Phosphorylated Tau 244–441	Promotes fibrillation [65]
	SOD1 A4V	Promotes fibrillation [65]
	Rabbit PrP	Hinders fibrillation [65]
	HEWL	Hinders fibrillation [65]
	β-lactoglobulin	Promotes fibrillation [63]
Dextran 100	α-lactalbumin	Increases lag phase, decreases elongation rate [12]
	Insulin, pH = 2.5	Decreases elongation rate [12]
	Insulin, pH = 7.5	Increases lag phase, decreases elongation rate [12]
	HEWL	Decreases elongation rate [12]
	α-synuclein	Decreases lag phase and elongation rate [12]
	Histone	Decreases lag phase [12]
Dextran 138	α-synuclein	Promotes fibrillation [42,48]
Dextran 200	HEWL	Promotes fibrillation [15]
	Insulin	Promotes fibrillation [15]
	PI3-SH3	Promotes fibrillation [15]
	α-synuclein	Promotes fibrillation [15]
	Insulin β Chain	Promotes fibrillation [15]
Dextran 250	Insulin, pH = 2.5	Decreases elongation rate [12]
	Insulin, pH = 7.5	Increases lag phase, decreases elongation rate [12]
	HEWL	Decreases elongation rate [12]
	α-synuclein	Decreases lag phase and elongation rate [12]
	Histone	Decreases lag phase, increases elongation rate [12]
	α-lactalbumin	Increases lag phase, decreases elongation rate [12]
Dextran 500	Insulin, pH = 2.5	Decreases elongation rate [12]
	Insulin, pH = 7.5	Increases lag phase, decreases elongation rate [12]
	HEWL	Decreases elongation rate [12]
	α-synuclein	Decreases lag phase and elongation rate [12]
	Histone	Decreases lag phase, increases elongation rate [12]
	α-lactalbumin	Increases lag phase, decreases elongation rate [12]
PEG 200	α-synuclein	Promotes fibrillation [42,48]
PEG 400	β-lactoglobulin	Promotes fibrillation [63]
	α-synuclein	Promotes fibrillation [42,48]
PEG 600	α-synuclein	Promotes fibrillation [42,48], Hinders fibrillation [66]
PEG 1000	α-synuclein	Increases lag time and fibrillation rate [66]
PEG 3350	α-synuclein	Promotes fibrillation [42]
PEG 3500	Insulin pH = 2	Promotes fibrillation [59]
	Insulin pH = 7.5	Hinders fibrillation [59]
	Histones pH = 2.5	Promotes fibrillation [59]
	Histones pH = 7.5	Hinders fibrillation [59]
	α-synuclein	Promotes fibrillation [42,59,60]
	α-lactalbumin	Promotes fibrillation [59]
PEG 4000	α-synuclein	Decreases lag time and fibrillation rate [66]
	Hemoglobin	Promotes fibrillation [67]
PEG 4400	α-synuclein	Promotes fibrillation [61]
	Insulin	Promotes fibrillation [61]
PEG 6000	Hemoglobin	Promotes fibrillation [67]
PEG 8000	β-lactoglobulin	Promotes fibrillation [63].
PEG 10,000	α-synuclein	Promotes fibrillation [42]
PEG 20,000	HEWL, unseeded	Hinders fibrillation [68]
	HEWL, seeded	Promotes fibrillation [68]
	Unphosphorylated Tau 244–372	Promotes fibrillation [65]
	SOD1 A4V	Promotes fibrillation [65]
	Rabbit PrP	Hinders fibrillation [65]
	β-lactoglobulin	Promotes fibrillation [63]
PEG 200,000	Insulin	Promotes Fibrillation [15]
HPC 100	Insulin pH = 2.5	Increases lag time and decreases elongation rate [12]
	Insulin pH = 7.5	Increases lag time and decreases elongation rate [12]
	α-synuclein	Increases lag time and decreases elongation rate [12]
	α-lactalbumin	Increases lag time and decreases elongation rate [12]
HPC 370	Insulin pH = 2.5	Increases lag time and decreases elongation rate [12]
	Insulin pH = 7.5	Increases lag time and decreases elongation rate [12]
	α-synuclein	Increases lag time and decreases elongation rate [12]
	α-lactalbumin	Increases lag time and decreases elongation rate [12]
HPC 1000	Insulin pH = 2.5	Increases lag time and decreases elongation rate [12]
	Insulin pH = 7.5	Increases lag time and decreases elongation rate [12]
	α-synuclein	Increases lag time and decreases elongation rate [12]
	α-lactalbumin	Increases lag time and decreases elongation rate [12]
UCON 5400	α-synuclein	Hinders fibrillation [61]
	Insulin	Hinders fibrillation [61]

## 5. Osmolytes

Osmolytes are small molecules that organisms develop in response to the stress induced by water loss [70]. These small organic molecules, which include amino acids and amino acid derivatives, sugars, urea, and methylamines [71], have been demonstrated to stabilize proteins in vitro [38] and in living cells [72]. Work by Serge Timasheff and coworkers demonstrates that stabilizing osmolytes operate by a preferential hydration method, where the osmolytes are excluded from the protein, resulting in hydration of the protein surface [73]. Native state stabilization, resulting from a combination of steric and chemical effects [74,75], was proposed to be a result of the native state being favored compared to the denatured state of the protein, as there was less surface area to be excluded from the osmolytes [76,77]. Bolen and coworkers referred to this as the “osmophobic effect.” This theory was further refined to include the repulsive interactions between stabilizing osmolytes and the protein backbone, that raise the energy of the denatured state relative to the native state [78]. Various studies have confirmed the unfavorable enthalpic interactions between the osmolytes and proteins for protein stability [38], and the kinetics of protein folding [39].

## 6. Osmolytes and Protein Fibrillation

In their 2010 *Journal of the American Chemical Society* publication [15], Dobson and coworkers studied the effects of an osmolyte, glucose, on the fibrillation of the monomeric globular proteins, lysozyme and insulin. The proteins lacking a well-defined tertiary structure were also considered, including the SH3 domain of the PI3-Kinase (PI3K-SH3), α-synuclein, and the β-domain of insulin at pH = 2. The results were striking, indicating that glucose has variable effects on the fibrillation of proteins. Specifically, the globular proteins with a compact native structure (lysozyme and insulin) saw their fibrillation hindered by the presence of 200 g/L glucose, while the fibrillation of the three natively unfolded proteins (SH3, α-synuclein, and β-chain of insulin) was accelerated. Dobson and coworkers connected this duality with the nature of the native state of the proteins in question. The globular proteins with a compact native state must unfold to aggregate. Conversely, the disordered proteins, PI3-SH3, α-synuclein, and the β chain of insulin, adopt native state conformations that are already extended. For these proteins, the aggregation-prone transition state is likely more compact, and therefore favored, in an environment crowded with osmolytes. However, the osmolytes hinder the fibrillation of globular proteins, which must adopt a more extended transition state before forming aggregates.

Several studies have since confirmed that the osmolytes promote the fibrillation of disordered proteins, such as α-synuclein, [59,62], and hinder the fibrillation of the globular proteins, including insulin [79,80,81], lysozyme [82,83,84], BSA [85], and the *T. thermophilus* ribosomal protein S6 [86]. However, a host of research groups have further explored the complex effects of osmolytes on protein fibrillation.

A recent study by Islam and colleagues explored the ability of sugars to protect α-lactalbumin (α-LA) from aggregation, using sucrose, its monomers, glucose and fructose, and a mixture of glucose and fructose as crowding agents [87]. Although kinetic studies were not employed, Thioflavin T and tryptophan fluorescence experiments, along with Rayleigh scattering and Dynamic Light Scattering (DLS), demonstrated that the sugar osmolytes reduced the amount of aggregates formed, suggesting that sugars have an inhibitory effect on α-LA fibrillation. Of all the solutions, glucose on its own is the least effective inhibitor, while the mixture of glucose and fructose is the most effective. The authors endeavored to explain this phenomenon through molecular docking simulations between sugars and several residues within α-LA. The docking experiments showed that the hydrogen bonds may occur between α-LA and sugars, indicating that weak interactions between the sugars and the protein, rather than solely excluded volume or viscosity effects, may influence the observed inhibition of fibril formation.

The work of the Bhat group has recently sought to understand the effect of polyol osmolytes on protein fibrillation, specifically, how the addition of -OH groups alters fibrillation [88,89]. The molecules utilized are ethylene glycol (2 -OH groups), glycerol (3), erythritol (4), xylitol (5), and sorbitol (6). The work of Roy and Bhat dug deeper into the effects of osmolytes in human γ-synuclein which, in the same way as α-synuclein, is intrinsically disordered [88]. Ethylene glycol promotes fibrillation concentrations lower than 4.5 M and suppresses fibrillation at concentrations greater than 4.5 M. Glycerol, the smallest polyol osmolyte, also suppresses fibrillation by increasing the lag time, decreasing the rate of fibrillation, and decreasing the overall number of fibrils. Erythritol and xylitol both increase the lag time with increasing concentration, but xylitol also decreases the rate of fibrillation. Finally, the largest polyol, sorbitol, increases the lag time at low concentrations, but decreases the lag time and the fibrillation rates at the high concentrations. The differing effects on γ-synuclein suggest that the influence of the osmolytes depends on the structure and number of -OH groups contained in the polyols. Specifically, the lag time decreases with the increased number of -OH groups. This trend arises from the degree of preferential exclusion, and whether the osmolyte preferentially stabilizes the monomer, fibril, or an intermediate along the fibrillation pathway. These results suggest that the relationship between the osmolytes and disordered proteins is more complex than initially proposed by Dobson and coworkers.

A subsequent study from the Bhat group expanded their exploration of the polyol osmolyte size effects to α- and β-synuclein [89]. While α-synuclein is fibrillation-prone, β-synuclein resists fibrillation, due to the lack of a non-amyloid β component (NAC) domain. Generally, polyol osmolytes promote α-synuclein fibrillation. Ethylene glycol induces a decrease in the lag time and an increase in the rate of fibrillation, which the authors attributed to favoring the nucleation of early-stage oligomers. The light scattering data indicated that smaller aggregates are formed in ethylene glycol, which was attributed to the high viscosity of the ethylene glycol solutions. The addition of another -OH group with glycerol alters the way the osmolyte modulated the fibrillation. From concentrations of 0.25 to 2 M, glycerol decreases the lag time and increases the fibrillation rate of a-synuclein. However, at high concentrations (>2 M), the mechanism changed— the lag time increases and the fibrillation rate decreases relative to the buffer, a trend observed previously with glycerol and α-synuclein [42].

The authors rationalized that at low concentrations of glycerol, preferential exclusion from the protein surface dominates, favoring the fibril over the disordered monomer. At higher concentrations, this effect is overtaken by the increasing viscosity of the solution. The fibrillation of α-synuclein is promoted in the presence of erythritol at all concentrations; the lag time decreases, and the apparent rate of fibrillation increases. Xylitol and Sorbitol, however, show non-monotonic effects, in the same way as glycerol, with an inflection point at 1.5 M. The xylitol decreases the lag time of the fibrillation with increasing fibrillation, while the apparent rate of fibrillation increased until 2 M, where it decreased, relative to the buffer. After an initial increase at 0.25 M, the sorbitol also decreased the lag time with increasing concentration and increased the fibrillation rate, with a slight decrease relative to the buffer at 1.5 M. Ultimately, the authors attributed these effects at high concentrations to an increase in the viscosity of the solution, as the viscosity increases with the concentration and number of -OH groups. These findings were further supported when the authors tracked the ANS-binding fluorescence intensity and light scattering as a function of the concentration; decreasing the scattering and ANS binding—an effect that increased with the concentration and number of the polyols. This indicated that the concentration of the fibrils formed decreased with the increasing concentration and size of the osmolyte, while SEM indicated that the fibrils formed exhibited a different morphology. Ultimately, the authors proposed a two-fold model for fibrillation in the presence of osmolytes, depending on concentration. At low concentrations, the monomers diffuse readily, and the formation of the fibrils is facilitated due to preferential exclusion. At high concentrations, where the viscosity is high, the monomers diffuse less readily, and shorter fibrils are favored, due to preferential exclusion, instead of longer ones.

Two papers compared the effects of stabilizing osmolytes and destabilizing osmolytes on the fibrillation of proteins. The N-terminal fragment of the *E.* *coli* hydrogenase maturation factor, HypF (HypF-N), a model amyloidogenic protein, was the subject of a study by Roy and colleagues [90], while insulin was explored by the Belfort group [91]. Consistent with the observations from Dobson’s study, insulin fibrillation is hindered by mono-, di-, and trisaccharide osmolytes via preferential exclusion and stabilization of the native protein; the lag times are increased and nucleation rates are decreased. As the size, and therefore potential for preferential exclusion, increases, so do the effects. The opposite trend, however, was seen with the destabilizing osmolytes urea and guanidinium HCl. The lag times are decreased, and the nucleation and fibrillation rates are increased. Similar results were observed for the model protein, HypF-N; the stabilizing osmolytes hinder fibrillation, likely through a stabilization of the native state. The one exception is proline, which promotes fibrillation. Where this study diverges from the other, however, is that, for HypF-N, unlike insulin, guanidinium HCl, and urea, *hindered* protein fibrillation, which was attributed to the interactions between the osmolytes and water.

Many studies have explored the effects of osmolytes on the small peptide hormones and model peptides and proteins (see e.g., [90,92,93,94,95,96] and references cited therein). Since these hormones and peptides do not fall into the categories delineated by Dobson and colleagues, they will be considered separately.

Harries and colleagues used a model peptide, termed MET16, which on its own forms a stable, monomeric B-hairpin but can also unfold and then aggregate into fibrils [92]. The fibrillation was measured in the presence of glycerol, sorbitol, and triethylene glycol, in addition to PEG 400 and 4000. The small molecule osmolytes slow fibrillation, while PEGs exert little to no effect. In addition, unlike the other studies, the presence of cosolutes was not found to affect the morphology or yield of fibrils. Of particular interest to the authors was that, while the sorbitol and triethylene glycol have a similar size, the effects of the sorbitol on MET16 fibrillation were greater. Although all of the cosolutes—polyols and PEGS—were found to operate via preferential hydration, they had varying effects on MET16, implicating the role of peptide sequence and soft interactions between peptide and crowder. Since MET16 must unfold to fibrillate, these results are compatible with Dobson’s findings and predictions for globular proteins.

**Table 2:** Effects of osmolytes and small molecules on protein fibrillation.

Cosolute	Test Protein	Effect
Glucose	HEWL	Hinders fibrillation [15]
	Insulin	Hinders fibrillation [15,91]
	PI3-SH3	Promotes fibrillation [15]
	α-synuclein	Promotes fibrillation [15]
	Insulin β Chain	Promotes fibrillation [15]
	3HmutWil	Hinders fibrillation [93]
	α-lactalbumin	Hinders fibrillation [87]
	Glucagon	No effect [96]
Sucrose	Insulin	Hinders fibrillation [79,91]
	3HmutWil	Hinders fibrillation [93]
	HypF-N	Hinders Fibrillation [90]
	α lactalbumin	Hinders fibrillation [87]
	S6	Hinders fibrillation [86]
	Human Tau Protein	Hinders fibrillation [64]
	Glucagon	No effect [96]
Fructose	α lactalbumin	Hinders fibrillation [87]
	Insulin	Hinders fibrillation [91]
Fructose + Sucrose	α-lactalbumin	Hinders fibrillation [87]
Trehalose	3HmutWil	Hinders fibrillation [93]
	HypF-N	Hinders Fibrillation [90]
	α-synuclein	Promotes fibrillation [97]
	Insulin	Hinders fibrillation [80,91]
	Glucagon	No effect [96]
Glycerol	α-synuclein	Promotes fibrillation [42]
	MET 16	Hinders fibrillation [92]
	HypF-N	Hinders Fibrillation [90]
	γ-synuclein	Hinders fibrillation [88]
	Glucagon	No effect {Citation}
	α-synuclein, 0.25–2 M	Promotes fibrillation [89]
	α-synuclein, 4.0–6.0 M	Hinders fibrillation [89]
Ethylene Glycol	α-synuclein	Promotes fibrillation [48,89]
	γ-synuclein (<4.5 M)	Hinders fibrillation [88]
	γ-synuclein (>4.5 M)	Promotes fibrillation [88]
Sorbitol	MET 16	Hinders fibrillation [92]
	Insulin	Hinders fibrillation [81]
	γ-synuclein	Reduces lag time, increases fibrillation rate [88]
	BSA	Hinders fibrillation [85]
	α-synuclein	Hinders fibrillation [89]
	Glucagon	No effect [96]
Triethylene Glycol	MET 16	No effect [92]
TMAO	α-synuclein	Promotes fibrillation [59,62]
	hIAPP	Hinders fibrillation [94]
	HypF-N	Hinders Fibrillation [90]
	HEWL	Hinders fibrillation [69,70]
Betaine	Insulin	Hinders fibrillation [80,81]
	IAPP	Hinders fibrillation [94]
	HypF-N	Hinders Fibrillation [90]
	Glucagon	Promotes fibrillation [96]
Glycine Betaine	BSA	Promotes Fibrillation [85]
	Glucagon	Promotes fibrillation [96]
Citrulline	Insulin	Hinders fibrillation [80,81]
Proline	Insulin	Hinders fibrillation [81]
	HypF-N	Promotes Fibrillation [90]
	HEWL	Hinders fibrillation [84]
	BSA	Hinders fibrillation [85]
Hydroxyproline	HEWL	Hinders fibrillation [84]
	BSA	Hinders fibrillation [85]
Sarcosine	HypF-N	Hinders Fibrillation [90]
	HEWL	Hinders fibrillation [84]
	BSA	Hinders fibrillation [85]
	Glucagon	Promotes fibrillation [96]
Urea	IAPP	Hinders fibrillation [94]
	HypF-N	Hinders Fibrillation [90]
	Insulin	Promotes fibrillation [91]
Erythritol	γ-synuclein	Hinders fibrillation [88]
	α-synuclein	Promotes fibrillation [89]
Xylitol	γ-synuclein	Promotes fibrillation [88]
	α-synuclein (<2 M)	Promotes fibrillation [89]
	α-synuclein (2 M)	Hinders fibrillation [89]
Maltose	Insulin	Hinders fibrillation [91]
Raffinose	Insulin	Hinders fibrillation [91]
Ectoine	PrP	Hinders fibrillation [95]
	Insulin	Hinders fibrillation [80]
	Glucagon	Promotes fibrillation [96]
Hydroxyectoine	PrP	No effect [95]
Taurine	Glucagon	Promotes fibrillation [96]
Ascorbic acid	HEWL	Hinders fibrillation [82]

HEWL, hen egg white lysozyme; PI3-SH3, Src-homology 3 domain of phosphatidyl-inositol-3-kinase; hIAPP, human islet amyloid polypeptide; SOD1, superoxide dismutase 1.

**Table 3:** Effects of protein crowders on protein fibrillation.

Cosolute	Test Protein	Effect
BSA	α-synuclein	Promotes fibrillation [42]
	hIAPP	Hinders fibrillation [69]
	α-lactalbumin	Promotes fibrillation [59]
HEWL	α-synuclein	Promotes fibrillation [42]
	hIAPP	Hinders fibrillation [69]

HEWL, hen egg white lysozyme; PI3-SH3, Src-homology 3 domain of phosphatidyl-inositol-3-kinase; hIAPP, human islet amyloid polypeptide; SOD1, superoxide dismutase 1.

Ueda and coworkers evaluated the effects of the sugar osmolytes, glucose, sucrose, and trehalose on the fibrillation of 3HmutWil, the peptide portion of a mutated, amyloid-prone light chain Wil of the Vλ6 protein. The unfolded version of this protein has been implicated in a monoclonal plasma cell disorder [93]. To mimic the fibrillation of the unfolded peptide, the fibrillation experiments were carried out at pH = 2, where 3HmutWil is unfolded. Under these conditions, the sucrose, glucose and trehalose increase the lag phase, hindering the 3HmutWil fibrillation. The sucrose had the greatest effect, trehalose was similar, while glucose was the least effective at hindering the fibrillation. However, when the pre-seeded fibrils were added to the sucrose, glucose and trehalose solutions, fibrillation was unaffected by the addition of the osmolytes. CD and 2D nuclear magnetic resonance (NMR) experiments in the presence of the sugars indicated that the 3HmutWil was refolded in the presence of sugars. These data combined indicate that the sugar osmolytes affect 3HmutWil fibrillation by stabilizing the compact native state, and do not influence the fibril elongation process. Ultimately, a lack of difference between the 2D NMR spectra, in the presence or absence of sugar, indicates that the structural changes are likely not due to direct interactions between the folded state of 3HmutWil and sugar osmolytes, but rather through preferential hydration of the native state.

Winter and colleagues investigated the effects of non-sugar osmolytes on the fibrillation of the islet amyloid polypeptide (IAPP), which is implicated in Type 2 diabetes [94]. The IAPP is unfolded in its native monomeric state but can form transient structures. The effects of the stabilizing osmolytes, TMAO and betaine, the destabilizing osmolyte urea, and the combinations of urea/betaine and urea/TMAO were considered. The lag phase of fibrillation is unaffected by the addition of 1 and 2 M TMAO and betaine, but the fibrillation rate decreases, while the sigmoidal shape of the curve is lost. The authors suggest that TMAO and betaine may stabilize smaller oligomers or protofibirils, rather than the monomer or longer fibril. The TMAO effects were observed to be stronger and more concentration-dependent than betaine. Interestingly, the addition of urea increased the lag phase of IAPP fibrillation in a concentration-dependent manner, suggesting that urea stabilizes the unfolded native state, delaying the fibrillation. The addition of TMAO to a solution of urea fully counteracts the delay induced by urea, while betaine counteracts the effect of urea by only a small amount. This suggests that the observed effects are due to interactions between the stabilizing and destabilizing cosolutes added, rather than interactions between the individual cosolutes and the peptide. The AFM imaging found that the morphology of fibrils was unchanged by the addition of cosolvents, pointing towards interactions with the native protein as the cause for any changes to fibrillation kinetics. Ultimately, the observed effects are attributed to preferential exclusion from the unfolded native state by TMAO and betaine, leading to small oligomers, while urea preferentially hydrates the unfolded native state, prolonging the lag phase. Although different mechanisms are adopted, both delay the fibrillation of an unfolded peptide, and ultimately contradict the effects observed by Dobson and coworkers for the unfolded proteins in the presence of sugar osmolytes.

Park and colleagues investigated the effects of four osmolytes on the fibrillation of residues 106–126 of the human prion peptide, PrPc [95]. A conformational change in this 20-residue peptide is implicated in the conversion of the non-fibrillation-prone prion protein, PrPc, into the fibrillation-prone version, PrPsc. This peptide contains a polar headgroup and a hydrophobic tail. The ability of the stress molecules to alter the fibrillation of this segment of PrP was explored. Ectoine, hydroxyectoine, mannosylglycerate, and mannosylglyceramide, which are produced by cells under stress conditions, were selected. All inhibit fibrillation, with ectoine and mannosylglyceramide showing the strongest concentration-dependent effects. Additionally, the cells treated with these stress molecules showed increased viability, compared to the untreated cells, pointing to the therapeutic potential of stress molecules against aggregation-based diseases. These two osmolytes were proposed to hinder fibrillation by preferential exclusion from the native protein. The other stress molecules likely operated via a different mechanism. Hydroxyectoine, which is more polar than ectoine due to an extra OH group, and mannosylglycerate, which has a negative charge and may interact with the negative head group, freed the hydrophobic tail to aggregate. Ultimately, the effects of the stress molecules are mechanism-dependent, and may vary based on the structure of the chosen molecule and test protein.

Otzen and colleagues considered the effects of a host of osmolytes—including polyols, amino acids, and methylamines—on the fibrillation of the hormone, glucagon [96]. The polyols had a minimal effect on glucagon fibrillation. The amino acids exhibited a decrease in the lag time of fibrillation, promoting fibrillation. The one exception was taurine, which increased the lag time, delaying fibrillation. The methylamines, specifically sarcosine and betaine, also decreased the lag time. Concentration-dependence was explored, but only sarcosine exhibited a noticeable concentration-dependent decrease in the lag time. CD was used to probe the lack of effects on the kinetics—to see if any possible differences arose in the fibril structure. The polyols did not affect the structure of the fibrils, but the amino acids and methylamines led to the production of a different class of fibril than was observed in the dilute solution. These observations led the authors to conclude that a blanket theory cannot be applied to osmolyte–protein systems; the effects vary, depending on the protein and osmolyte chosen for the study.

## 7. Protein Crowders

Many studies have explored the effects of synthetic polymers and osmolytes on the fibrillation of proteins [48,64,68,81,86,87]. Some have even expanded the study to physiologically relevant conditions, by exploring the effects of these crowders on cell viability and toxicity [67,95]. However, while the osmolytes do populate cells under stress conditions, the synthetic polymers are not naturally found in cells. As the cell contains a high concentration of proteins in the cytoplasm, the proteins can serve as physiologically relevant crowding agents, and can inform our understanding of how fibrillation occurs in the cell. Hen egg white lysozyme and BSA are commonly used as the crowding agents, due to their durability and ease of purchase [38,39,48]. However, working with proteins as crowding agents poses complications, as some of the proteins used as crowding agents can themselves fibrillate [82,83,84,85,98], and cannot be used with techniques commonly used to assess fibril formation and morphology, such as CD, as the protein crowder’s signal would interfere with that of the test protein [69]. Only a few studies, therefore, have endeavored to test the effects of protein crowders on protein fibrillation. As seen with both polymer and osmolyte studies, the effects are not uniform.

## 8. Protein Crowders and Fibrillation

Uversky and colleagues explored the effects of the protein crowders, BSA and lysozyme, on the fibrillation of α-synuclein [42,48]. Both were found to accelerate α-synuclein fibrillation by decreasing the lag time and increasing the acceleration rate, even at a low concentration (60 g/L BSA, 50 g/L lysozyme). In fact, both were more effective at promoting α-synuclein fibrillation, even at lower g/L concentrations, than the chosen synthetic polymers. The subsequent studies on the effect of BSA at 30 g/L on the fibrillation of another disordered protein, bovine s-carboxymethyl-a-lactalbumin, confirmed that the BSA promotes the fibrillation of disordered proteins [59]. Interestingly, the authors addressed BSA and lysozyme as inert protein crowders. At the pH chosen, the α-synuclein is negatively charged, while the lysozyme is positively charged (+8), and the BSA is negatively charged (−17). Under these conditions, the BSA exhibited stronger effects than the lysozyme. The authors eliminated any charge–charge contribution, and instead, only considered the effects of excluded volume. However, later studies have demonstrated that charge–charge attractions between test proteins and protein crowders can destabilize the native state of a protein, while repulsions can also destabilize a protein, but to a lesser extent [38]. These revelations indicate that the effects of chemical interactions between proteins, especially in the context of living cells, need to be further explored and analyzed.

The opposite effects were seen when the BSA and lysozyme were used as crowding agents in the study of the fibrillation of another natively unfolded peptide, IAPP; indicating that crowding by proteins must be more complicated than excluded volume alone [69]. In this analysis, Winter and colleagues did consider the chemical interactions that might occur between the protein crowders and IAPP, but not between the synthetic polymers and IAPP. Both the BSA and lysozyme hindered IAPP fibrillation in a concentration-dependent matter and decreased the number of fibrils; more drastic effects were seen with the lysozyme than with the BSA. This hindrance of fibrillation was also seen with synthetic polymer crowders. Despite the similar effects in terms of kinetics, the authors focused on the difference between the mechanisms adopted by the proteins and synthetic crowders by imaging the aggregates formed in the presence of both classes, using AFM. The fibrils that formed in the presence of Ficoll and dextran maintained the same morphology as the fibrils formed in a dilute solution. In the presence of the BSA and lysozyme, globular IAPP monomers and oligomers are observed—suggesting that the protein crowders stabilize the off-pathway species, hindering fibrillation. The authors concluded that the two types of crowders both delay fibrillation, by stabilizing the off-pathway monomers and oligomers. However, the protein crowders showed more pronounced effects at lower concentrations, suggesting weak chemical interactions dominated these conditions, while excluded volume and viscosity and diffusion effects were probably the main contributors to the effects seen with synthetic polymers.

## 9. Summary and Conclusions

The effect of macromolecular crowding on protein fibrillation protein depends on the class of crowder studied. Stabilizing osmolytes act mainly on the native state of the globular and oligomeric proteins, increasing the equilibrium thermodynamic stability. This decreases the population of non-native states, which may explain the commonly seen reduction in fibrillation rates. The effects on the native state outweigh the effects further down the pathway, except in two cases: the pairing of glycine betaine with BSA, and of proline with HypF-N. In these instances, the osmolytes favor fibrillation, probably by stabilizing the intermediates or lowering barriers along the fibrillation pathway. Destabilizing osmolytes, such as urea, may have the opposite effect at low to moderate concentrations. However, urea is also a denaturant, such that high concentrations favor complete unfolding and disfavor fibrillation.

In contrast to the globular proteins, the osmolytes exhibit variable effects on the fibrillation of disordered proteins and small peptides. Here, native state stabilization is not important; instead, the alteration in the free energy of the intermediates and barriers along the fibrillation pathway takes precedence.

Large, synthetic polymers, such as dextran or Ficoll, increase the fibrillation rates in disordered proteins and small peptides. The size and concentration of these crowders reduces the available solution volume, and accelerates the association steps that are key to fibrillation. For globular and oligomeric proteins, the results are more varied.

The protein crowders promote fibrillation in some cases and hinder it in others. This variability is likely due to weak, nonspecific protein–protein interactions that become noticeable at the high concentrations used in these experiments.

Overall, the large number of effects at the molecular level make it difficult to conclude much about the common fibrillation mechanisms using in vitro crowding agents. All come with caveats, and it is difficult to recommend an ideal certain type. Ultimately, in vitro measurements alone are of limited value. For more than 40 years, scientists have characterized the effects of the cellular interior on protein function. The advances in technology, such as in-cell NMR [9,99], single-cell mass spectrometry [100], fluorescence [8,10,101,102], FRET [103] and flow cytometry [104], electron microscopy [5,6,7], and cryo-electron tomography (Cryo-ET), allow scientists to characterize the protein and the protein aggregate structure and function in cells. Cryo-ET is particularly effective at structural characterization of the neurotoxic aggregates [105,106,107]. These advances, and others that will come, empower scientists to expand upon the efforts discussed and characterize all of the aspects of protein aggregation, including kinetics, in living cells.

## Data Availability

Not applicable.

## Supplementary Materials

The following supporting information can be downloaded at: https://www.mdpi.com/article/10.3390/biom12070950/s1, Table S1: Table 1 organized by protein; Table S2: Table 2 organized by protein.
