# Supplementary material for: Protein Fibrillation under Crowded Conditions
Source: Biomolecules. 2022 Jul 6;12(7):950. doi: 10.3390/biom12070950 (PMC9312947; doi:10.3390/biom12070950)
Supplement: Supplementary file 1 [file biomolecules-12-00950-s001.zip › biomolecules-1778678-supplementary.pdf]

biomolecules

MDPI

Review

Supplementary Information For: Protein Fibrillation Under Crowded Conditions

Annelise H. Gorensek-Benitez [1], Bryan Kirk [2] and Jeffrey Myers [3,*]

[1] Dept. of Chemistry and Biochemistry, Colorado College, Colorado Springs, CO 80903
[2] Dept. of Biology, Davidson College, Davidson, NC 28035
[3] Dept. of Chemistry, Davidson College, Davidson, NC 28035
* Correspondence: agorensekbenitez@coloradocollege.edu or jemyers@davidson.edu

Abstract:

Protein amyloid fibrils have widespread implications for human health. Over the last twenty years, fibrillation has been studied using a variety of crowding agents to mimic the packed interior of cells or to probe the mechanisms and pathways of the process. We tabulate and review these results by considering three classes of crowding agent: synthetic polymers, osmolytes and other small molecules, and globular proteins. While some patterns are observable for certain crowding agents, the results are highly variable and often depend on the specific pairing of crowder and fibrillating protein.

Keywords: Aggregation, amyloid fibril, excluded volume, molecular crowding, neurodegenerative disease, molecular crowding, osmolyte, polyol, protein fibrillation, proteopathy, synthetic polymer, viscosity

Table S1. Abbreviations and average molecular weights for polymers referenced in the text.

**Synthetic Polymer**	**Numerical Descriptor**	**Average Molecular weight (Da)**
PEG-	200	200
	400	400
	600	600
	1000	1000
	2000	2000
	3350	3350
	3500	3500
	4000	4000
	4400	4400
	6000	6000
	8000	8000
	10,000	10,000
	20,000	20,000
	200,000	200,000
Ficoll-	70	70,000
	400	400,000
Dextran-	70	70,000
	100	100,000
	138	138,000
	200	200,000
	250	250,000
	500	500,000
HPC	100	100,000
	370	370,000
	1000	1,000,000
UCON	5400	5400

Footnotes: PEG, polyethylene glycol; HPC, hydroxypropylcellulose; UCON is a trade name.

Table S2. Effects of macromolecular crowders in intrinsically disordered proteins (IDPs). Table is arranged alphabetically by protein.

Test Protein	Cosolute	Effect
α-synuclein	BSA	Promotes fibrillation [1]
	Dextran 70	Promotes fibrillation [2]
	Dextran 100	Decreases lag phase and elongation rate [3]
	Dextran 138	Promotes fibrillation [1,4]
	Dextran 200	Promotes fibrillation [5]
	Dextran 250	Decreases lag phase and elongation rate [3]
	Dextran 500	Decreases lag phase and elongation rate [3]
	Erythritol	Promotes fibrillation [6]
	Ethylene Glycol	Promotes fibrillation [4] [6]
	Ficoll 70	Promotes fibrillation [1,2,4,7]
	Ficoll 400	Promotes fibrillation [1,4,7]
	Glucose	Promotes fibrillation [5]
	Glycerol	Promotes fibrillation [1]
	Glycerol (0.25-2 M)	Promotes fibrillation [6]
	Glycerol (4.0-6.0 M)	Hinders fibrillation [6]
	HEWL	Promotes fibrillation [1]
	HPC 100	Increases lag time and decreases elongation rate [3]
	HPC 370	Increases lag time and decreases elongation rate [3]
	HPC 1000	Increases lag time and decreases elongation rate [3]
	PEG 200	Promotes fibrillation [1,4]
	PEG 400	Promotes fibrillation [1,4]
	PEG 600	Promotes fibrillation [1,4], Hinders fibrillation [8]
	PEG 1000	Increases lag time and fibrillation rate [8]
	PEG 2000	Promotes fibrillation [1,2,7]
	PEG 4000	Decreases lag time and fibrillation rate [8]
	PEG 4400	Promotes fibrillation [9]
	Sorbitol	Hinders fibrillation [6]
	TMAO	Promotes fibrillation [7,10]
	Trehalose	Promotes fibrillation [11]
	UCON 5400	Hinders fibrillation [9]
	Xylitol (< 2 M)	Promotes fibrillation [6]
	Xylitol (2 M)	Hinders fibrillation [6]
γ-synuclein	Erythritol	Hinders fibrillation [12]
	Ethylene Glycol (< 4.5 M)	Hinders fibrillation [12]

	Ethylene Glycol (> 4.5 M)	Promotes fibrillation [12]
	Glycerol	Hinders fibrillation [12]
	Sorbitol	Reduce lag time, increases fibrillation rate [12]
	Xylitol	Promotes fibrillation [12]
Histone	Dextran 100,000	Decreases lag phase [3]
	Dextran 250,000	Decreases lag phase, increases elongation rate [3]
	Dextran 500,000	Decreases lag phase, increases elongation rate [3]
Histone, pH = 2	PEG 3500	Promotes fibrillation {Citation}
Histone, pH= 7.5	PEG 3500	Hinders fibrillation
Tau Protein	Dextran 70 (50-100 g/L)	Promotes fibrillation [13]
	Dextran 70 (100 g/L)	Hinders fibrillation [13]
	Ficoll 70	Promotes fibrillation [13]
	Sucrose	Hinders fibrillation [13]
Phosphorylated Tau 244-441	Dextran 70	Promotes fibrillation [14]
	Ficoll 70	Promotes Fibrillation [14]
Unphosphorylated Tau 244-372	Dextran 70	Promotes fibrillation [14]
	Ficoll 70	Promotes Fibrillation [14]
	PEG 20,000	Promotes fibrillation [14]
MET 16	Glycerol	Hinders fibrillation [15]
	Sorbitol	Hinders fibrillation [15]
	Triethylene Glycol	No effect [15]
PI3-SH3	Dextran 200	Promotes fibrillation [5]
	Glucose	Promotes fibrillation [5]
Vλ6 (3HmutWil mutant)	Glucose	Hinders fibrillation [16]
	Sucrose	Hinders fibrillation [16]
	Trehalose	Hinders fibrillation [16]

Footnotes. HEWL, hen egg white lysozyme; PrP, prion protein; HypF-N, N-terminal domain of the *E. coli* HypF protein; PI3-SH3, Src-homology 3 domain of phosphatidyl-inositol-3-kinase; PEG, polyethylene glycol; HPC, hydroxypropylcellulose; UCON is a trade name.

Table S3. Effects of crowding on monomeric globular proteins. Data are arranged by protein name in alphabetical order.

Test Protein	Cosolute	Effect
β-lactoglobulin	Dextran 70	Promotes fibrillation [17]
	PEG 400	Promotes fibrillation [17]
	PEG 8000	Promotes fibrillation [17]
	PEG 20,000	Promotes fibrillation [17]
Bovine Carbonic Anhydrase	Dextran 70	Hinders fibrillation [18]
	Ficoll 70	Hinders fibrillation [18]
BSA	Glycine Betaine	Promotes Fibrillation [19]
	Hydroxyproline	Hinders fibrillation [19]
	Proline	Hinders fibrillation [19]
	Sarcosine	Hinders fibrillation [19]
	Sorbitol	Hinders fibrillation [19]
HEWL	Ascorbic acid	Hinders fibrillation [20]
	Dextran 70	Hinders fibrillation [14]
	Dextran 100	Decreases elongation rate [3]
	Dextran 200	Promotes fibrillation [5]
	Dextran 250	Decreases elongation rate [3]
	Dextran 500	Decreases elongation rate [3]
	Ficoll 70	Hinders Fibrillation [14]
	Glucose	Hinders fibrillation [5]
	Hydroxyproline	Hinders fibrillation [21]
	Proline	Hinders fibrillation [21]
	Sarcosine	Hinders fibrillation [21]
	TMAO	Promotes fibrillation [7,10]
HEWL, seeded	PEG 20,000	Promotes fibrillation [22]
HEWL, unseeded	PEG 20,000	Hinders fibrillation [22]
HypF-N	Betaine	Hinders Fibrillation [23]
	Glycerol	Hinders Fibrillation [23]
	Proline	Promotes Fibrillation [23]
	Sarcosine	Hinders Fibrillation [23]
	Sucrose	Hinders Fibrillation [23]
	TMAO	Hinders Fibrillation [23]
	Trehalose	Hinders Fibrillation [23]
	Urea	Hinders Fibrillation [23]
PrP	Ectoine	Hinders fibrillation [24]
	Hydroxyectoine	No effect [24]

Human PrP	Ficoll 70	Promotes Fibrillation [14]
	Ficoll 400	Promotes Fibrillation [14]
Human PrP D178N	Ficoll 70	Promotes Fibrillation [14]
	Ficoll 400	Promotes Fibrillation [14]
Human PrP E196K	Ficoll 70	Promotes Fibrillation [14]
	Ficoll 400	Promotes Fibrillation [14]
Rabbit PrP	Dextran 70	Hinders fibrillation [14]
	Ficoll 70	Hinders fibrillation [14]
	PEG 20,000	Hinders fibrillation [14]
	PEG 20,000	Hinders fibrillation [14]

Footnotes. HEWL, hen egg white lysozyme; PrP, prion protein; HypF-N, N-terminal domain of the *E. coli* HypF protein; PI3-SH3, Src-homology 3 domain of phosphatidyl-inositol-3-kinase; PEG, polyethylene glycol; HPC, hydroxypropylcellulose; UCON is a trade name.

Table S4. Effects of crowding on oligomeric globular proteins. Data are arranged by protein name in alphabetical order.

Protein	Cosolute	Effect
α-lactalbumin	BSA	Promotes fibrillation [1]
	Dextran 100	Increases lag phase, decreases elongation rate [3]
	Dextran 250	Increases lag phase, decreases elongation rate [3]
	Ficoll 70	Promotes Fibrillation [7]
	Fructose	Hinders fibrillation [25]
	Fructose + Sucrose	Hinders fibrillation [25]
	Glucose	Hinders fibrillation [25]
	HPC 100	Increases lag time and decreases elongation rate [3]
	HPC 370	Increases lag time and decreases elongation rate [3]
	HPC 1000	Increases lag time and decreases elongation rate [3]
	PEG 3500	Promotes fibrillation [7]
	Sucrose	Hinders fibrillation [25]
Hemoglobin	Dextran 70	Promotes fibrillation [26]
	PEG 4000	Promotes fibrillation [26]
	PEG 6000	Promotes fibrillation [26]
Insulin	Betaine	Hinders fibrillation [27,28]
	Citrulline	Hinders fibrillation [27,28]
	Dextran	Promotes fibrillation [5]
	Ectoine	Hinders fibrillation [27]
	Fructose	Hinders fibrillation [29]
	Glucose	Hinders fibrillation [5] [29]
	Maltose	Hinders fibrillation [29]
	PEG 4400	Promotes fibrillation [9]
	Proline	Hinders fibrillation [28]
	Raffinose	Hinders fibrillation [29]
	Sorbitol	Hinders fibrillation [28]
	Sucrose	Hinders fibrillation [30] [29]
	Trehalose	Hinders fibrillation [27,29]
	UCON 5400	Hinders fibrillation [9]
	Urea	Promotes fibrillation [29]
Insulin, pH = 2	Ficoll 70	Promotes fibrillation [7]
	PEG 2000	Promotes fibrillation [7]
Insulin, pH = 2.5	Dextran 100	Decreases elongation rate [3]
	Dextran 250	Decreases elongation rate [3]
	Dextran 500	Decreases elongation rate [3]

	HPC 100	Increases lag time and decreases elongation rate [3]
	HPC 370	Increases lag time and decreases elongation rate [3]
	HPC 1000	Increases lag time and decreases elongation rate [3]
Insulin, pH = 7.5	Dextran 100	Increases lag phase, decreases elongation rate [3]
	Dextran 250	Increases lag phase, decreases elongation rate [3]
	Dextran 500	Increases lag phase, decreases elongation [3]
	Ficoll 70	Hinders fibrillation [7]
	HPC 100	Increases lag time and decreases elongation rate [3]
	HPC 370	Increases lag time and decreases elongation rate [3]
	HPC 1000	Increases lag time and decreases elongation rate
SOD1 A4V	Dextran 70	Promotes fibrillation [14]
	PEG 20,000	Promotes fibrillation [14]

Footnotes. SOD1, superoxide dismutase 1; PEG, polyethylene glycol; HPC, hydroxypropylcellulose; UCON is a trade name.

Table S5. Effects of crowding on small peptide hormones. Data are arranged by protein name in alphabetical order.

Protein	Cosolute	Effect
Glucagon	Betaine	Promotes fibrillation [31]
	Ectoine	Promotes fibrillation [31]
	Glucose	No effect [31]
	Glycerol	No effect [31]
	Glycine Betaine	Promotes fibrillation [31]
	Sarcosine	Promotes fibrillation [31]
	Sorbitol	No effect [31]
	Sucrose	No effect [31]
	Taurine	Promotes fibrillation [31]
	Trehalose	No effect [31]
hIAPP	Betaine	Hinders fibrillation [32]
	BSA	Hinders fibrillation [33]
	Ficoll 70,000	No effect [33]
	HEWL	Hinders fibrillation [33]
	TMAO	Hinders fibrillation [32]
	Urea	Hinders fibrillation [32]
	Dextran 70, 10-20%	No effect [33]
	Dextran 70, 30-40%	Hinders fibrillation [33]
Insulin β Chain	Dextran 200	Promotes fibrillation [5]
	Glucose	Promotes fibrillation [5]

Footnotes. hIAPP, human islet amyloid polypeptide; PEG, polyethylene glycol; HPC, hydroxypropylcellulose; UCON is a trade name.

References

1. Munishkina, Larissa A.; Cooper, E.M.; Uversky, V.N.; Fink, Anthony L. The Effect of Macromolecular Crowding on Protein Aggregation and Amyloid Fibril Formation. *Journal of Molecular Recognition* **2004**, *17*, 456–464.
2. Shtilerman, M.D.; Ding, T.T.; Lansbury, P.T.J. Molecular Crowding Accelerates Fibrillization of Alpha-Synuclein: Could an Increase in the Cytoplasmic Protein Concentration Induce Parkinson's Disease? *Biochemistry* **2002**, *41*, 3855–3860, doi:10.1021/bi0120906.
3. Breydo, L.; Reddy, K.D.; Piai, A.; Felli, I.C.; Pierattelli, R.; Uversky, V.N. The Crowd You're in with: Effects of Different Types of Crowding Agents on Protein Aggregation. *Biochim Biophys Acta (BBA) - Proteins and Proteomics* **2014**, *1844*, 346–357, doi:10.1016/j.bbapap.2013.11.004.

4. Uversky, V.N.; C. Accelerated α-Synuclein Fibrillation in Crowded Milieu. *FEBS Letters* **2001**, *515*, 99–103.
5. White, D.A.; B. Protein Aggregation in Crowded Environments. *Journal of the American Chemical Society* **2010**, *132*, 5170–5175.
6. Verma, G.; Singh, P.; Bhat, R. Disorder under Stress: Role of Polyol Osmolytes in Modulating Fibrillation and Aggregation of Intrinsically Disordered Proteins. *Biophysical Chemistry* **2020**, *264*, 106422, doi:10.1016/j.bpc.2020.106422.
7. Munishkina, L.A.; Ahmad, A.; Fink, A.L.; Uversky, V.N. Guiding Protein Aggregation with Macromolecular Crowding. *Biochemistry* **2008**, *47*, 8993–9006, doi:10.1021/bi8008399.
8. Biswas, S.; Bhadra, A.; Lakhera, S.; Soni, M.; Panuganti, V.; Jain, S.; Roy, I. Molecular Crowding Accelerates Aggregation of α-Synuclein by Altering Its Folding Pathway. *Eur Biophys J* **2021**, *50*, 59–67, doi:10.1007/s00249-020-01486-1.
9. Breydo, L.; Sales, A.E.; Frege, T.; Howell, M.C.; Zaslavsky, B.Y.; Uversky, V.N. Effects of Polymer Hydrophobicity on Protein Structure and Aggregation Kinetics in Crowded Milieu. *Biochemistry* **2015**, *54*, 2957–2966, doi:10.1021/acs.biochem.5b00116.
10. Uversky, V.N.; Li, J.; Fink, A.L. Trimethylamine-N-Oxide-Induced Folding of α-Synuclein. *FEBS Letters* **2001**, *509*, 31–35, doi:10.1016/S0014-5793(01)03121-0.
11. Naik, V.; Kardani, J.; Roy, I. Trehalose-Induced Structural Transition Accelerates Aggregation of α-Synuclein. *Molecular Biotechnology* **2016**, *58*, 251–255, doi:10.1007/s12033-016-9923-4.
12. Roy, S.; Bhat, R. Effect of Polyols on the Structure and Aggregation of Recombinant Human γ-Synuclein, an Intrinsically Disordered Protein. *Biochimica et Biophysica Acta (BBA) - Proteins and Proteomics* **2018**, *1866*, 1029–1042, doi:10.1016/j.bbapap.2018.07.003.
13. Wu, Y.; Teng, N.; Li, S. Effects of Macromolecular Crowding and Osmolyte on Human Tau Fibrillation. *International Journal of Biological Macromolecules* **2016**, *90*, 27–36, doi:10.1016/j.ijbiomac.2015.11.091.
14. Ma, Q.; Fan, J.-B.; Zhou, Z.; Zhou, B.-R.; Meng, S.-R.; Hu, J.-Y.; Chen, J.; Liang, Y. The Contrasting Effect of Macromolecular Crowding on Amyloid Fibril Formation. *PLoS One* **2012**, *7*, e36288, doi:10.1371/journal.pone.0036288.
15. Sukenik, S.P., Regina; Ziserman, Lior; Danino, Dganit; Friedler, Assaf; Harries Daniel Crowding Alone Cannot Account for Cosolute Effect on Amyloid Aggregation. *PLoS ONE* **2011**, *6*, e15608.
16. Abe, M.; Abe, Y.; Ohkuri, T.; Mishima, T.; Monji, A.; Kanba, S.; Ueda, T. Mechanism for Retardation of Amyloid Fibril Formation by Sugars in Vλ6 Protein. *Protein Sci* **2013**, *22*, 467–474, doi:10.1002/pro.2228.
17. Ma, B.; Xie, J.; Wei, L.; Li, W. Macromolecular Crowding Modulates the Kinetics and Morphology of Amyloid Self-Assembly by β-Lactoglobulin. *Int J Biol Macromol* **2013**, *53*, 82–87, doi:10.1016/j.ijbiomac.2012.11.008.
18. Mittal, S.; Singh, L.R. Macromolecular Crowding Decelerates Aggregation of a β-Rich Protein, Bovine Carbonic Anyhdrase: A Case Study. *J. Biochem.* **2014**, *156*, 273–282.
19. Dasgupta, M.; Kishore, N. Selective Inhibition of Aggregation/Fibrillation of Bovine Serum Albumin by Osmolytes: Mechanistic and Energetics Insights. *PLoS One* **2017**, *12*, e0172208, doi:10.1371/journal.pone.0172208.
20. Patel, P.; Parmar, K.; Patel, D.; Kumar, S.; Trivedi, M.; Das, M. Inhibition of Amyloid Fibril Formation of Lysozyme by Ascorbic Acid and a Probable Mechanism of Action. *Int J Biol Macromol* **2018**, *114*, 666–678, doi:10.1016/j.ijbiomac.2018.03.152.
21. Choudhary, S.; Kishore, N. Addressing Mechanism of Fibrillization/Aggregation and Its Prevention in Presence of Osmolytes: Spectroscopic and Calorimetric Approach. *PLOS ONE* **2014**, *9*, e104600, doi:10.1371/journal.pone.0104600.

22. Kong, L.-X.Z. Effects of Seeding on Lysozyme Amyloid Fibrillation in the Presence of Epigallocatchin and Polyethylene Glycol. *Biochemistry (Moscow)* **2017**, *82*, 266–279.
23. Bhavsar, R.D.; Prasad, S.; Roy, I. Effect of Osmolytes on the Fibrillation of HypF-N. *Biochimie* **2013**, *95*, 2190–2193, doi:10.1016/j.biochi.2013.07.019.
24. Kanapathipillai, M.; Ku, S.H.; Girigoswami, K.; Park, C.B. Small Stress Molecules Inhibit Aggregation and Neurotoxicity of Prion Peptide 106-126. *Biochem Biophys Res Commun* **2008**, *365*, 808–813, doi:10.1016/j.bbrc.2007.11.074.
25. Bashir, S.; Shamsi, A.; Ahmad, F.; Hassan, Md.I.; Kamal, M.A.; Islam, A. Biophysical Elucidation of Fibrillation Inhibition by Sugar Osmolytes in α-Lactalbumin: Multispectroscopic and Molecular Docking Approaches. *ACS Omega* **2020**, *5*, 26871–26882, doi:10.1021/acsomega.0c04062.
26. Siddiqui, G.A.; Naeem, A. The Contrasting Effect of Macromolecular Crowding and Confinement on Fibril Formation of Globular Protein: Underlying Cause of Proteopathies. *Journal of Molecular Liquids* **2021**, *322*, 114602, doi:10.1016/j.molliq.2020.114602.
27. Arora, A.; Ha, C.; Park, C.B. Inhibition of Insulin Amyloid Formation by Small Stress Molecules. *FEBS Lett* **2004**, *564*, 121–125, doi:10.1016/S0014-5793(04)00326-6.
28. Choudhary, S.; Kishore, N.; Hosur, R.V. Inhibition of Insulin Fibrilation by Osmolytes: Mechanistic Insights. *Scientific Reports* **2015**, *5*, 17599.
29. Nayak, A.; Lee, C.-C.; McRae, G.J.; Belfort, G. Osmolyte Controlled Fibrillation Kinetics of Insulin: New Insight into Fibrillation Using the Preferential Exclusion Principle. *Biotechnol Prog* **2009**, *25*, 1508–1514, doi:10.1002/btpr.255.
30. Marasini, C.F. Sucrose Modulates Insulin Amyloid-like Fibril Formation: Effect on the Aggregation Mechanism and Fibril Morphology. *RSC Advances* **2017**, *7*, 10487–10493.
31. Macchi, F.E., Maike; Kiefer, Hans; Otzen, Daniel E. The Effect of Osmolytes on Protein Fibrillation. *International Journal of Biological Macromolecules* **2012**, *13*, 3801–3819.
32. Seeliger, J.; Estel, K.; Erwin, N.; Winter, R. Cosolvent Effects on the Fibrillation Reaction of Human IAPP. *Phys Chem Chem Phys* **2013**, *15*, 8902–8907, doi:10.1039/c3cp44412k.
33. Seeliger, J.; Werkmüller, A.; Winter, R. Macromolecular Crowding as Suppressor of Human IAPP Fibril Formation and Cytotoxicity. *PLoS ONE* **2013**, *8*, e69652.
